# Interleukin-6 as a prognostic biomarker of clinical outcomes after traumatic brain injury: a systematic review

**DOI:** 10.1007/s10143-022-01827-y

**Published:** 2022-07-06

**Authors:** Setthasorn Zhi Yang Ooi, Robert James Spencer, Megan Hodgson, Samay Mehta, Nicholas Lloyd Phillips, Gwilym Preest, Susruta Manivannan, Matt P Wise, James Galea, Malik Zaben

**Affiliations:** 1 Cardiff University School of Medicine, Heath Park, Cardiff, UK; 2 Brain Research and Intracranial Neurotherapeutics (BRAIN) Unit, Neuroscience and Mental Health Innovation Institute, Cardiff University, Cardiff, UK; 3 Department of Neurosurgery, University Hospital of Wales, Cardiff, UK; 4 University of Birmingham Medical School, Birmingham, UK; 5 Princess of Wales Hospital, Bridgend, UK; 6 Department of Neurosurgery, Southampton General Hospital, Southampton, UK; 7 Adult Critical Care, University Hospital of Wales, Cardiff, UK

**Keywords:** Traumatic brain injury, Neuro-inflammation, Interleukin-6, Biomarker, Prognostication

## Abstract

Traumatic brain injury (TBI) is a major cause of mortality and morbidity worldwide. There are currently no early biomarkers for prognosis in routine clinical use. Interleukin-6 (IL-6) is a potential biomarker in the context of the established role of neuroinflammation in TBI recovery. Therefore, a systematic review of the literature was performed to assess and summarise the evidence for IL-6 secretion representing a useful biomarker for clinical outcomes. A multi-database literature search between January 1946 and July 2021 was performed. Studies were included if they reported adult TBI patients with IL-6 concentration in serum, cerebrospinal fluid (CSF) and/or brain parenchyma analysed with respect to functional outcome and/or mortality. A synthesis without meta-analysis is reported. Fifteen studies were included, reporting 699 patients. Most patients were male (71.7%), and the pooled mean age was 40.8 years; 78.1% sustained severe TBI. Eleven studies reported IL-6 levels in serum, six in CSF and one in the parenchyma. Five studies on serum demonstrated higher IL-6 concentrations were associated with poorer outcomes, and five showed no signification association. In CSF studies, one found higher IL-6 levels were associated with poorer outcomes, one found them to predict better outcomes and three found no association. Greater parenchymal IL-6 was associated with better outcomes. Despite some inconsistency in findings, it appears that exaggerated IL-6 secretion predicts poor outcomes after TBI. Future efforts require standardisation of IL-6 measurement practices as well as assessment of the importance of IL-6 concentration dynamics with respect to clinical outcomes, ideally within large prospective studies. Prospero registration number: CRD42021271200

**Supplementary Information:**

The online version contains supplementary material available at 10.1007/s10143-022-01827-y.

## Introduction

Traumatic brain injury (TBI) is a major cause of morbidity and mortality worldwide [1, 2]. The physical and cognitive disability resulting from TBI has a significant economic burden given that it disproportionately affects individuals of working age [1, 3]. Epidemiological studies have demonstrated limited improvements in mortality amongst TBI patients since 1990 [4], partly due to a paucity of effective pharmacological treatments. Moreover, accurate prognostication remains elusive due to a lack of predictive models, with the current most reliable predictor of the outcome being the Glasgow Coma Scale (GCS), which was developed over 40 years ago [5, 6].

A neuroinflammatory response to TBI is well recognised in the literature, and the degree and type of neuroinflammation have been shown to affect neurogenesis and functional recovery in laboratory-based models [7–9]. The inflammatory microenvironment after TBI is generated by neuronal disruption resulting in the release of damage-associated molecular patterns (DAMPs), causing the secretion of cytokines that recruit both local microglia and circulating macrophages [10]. The release of both pro- and anti-inflammatory cytokines occurs after TBI; however, the contribution of each in the progression of secondary brain injury and functional recovery is yet to be clearly defined. Amongst several inflammatory cytokines released post-injury, interleukin-6 (IL-6) is a key protein released by microglia, astrocytes and neurons post-TBI [11, 12]. At a cellular level, IL-6 has been implicated in promoting neuronal differentiation and survival post-injury via several mechanisms including tumour necrosis factor (TNF-alpha) inhibition, nerve growth factor (NGF) synthesis and modulating *N*-methyl-d-aspartate receptor (NMDAr)-mediated excitotoxicity [10].

Whilst IL-6 is often undetectable under physiological conditions in the brain, its acute release in response to injury is widely recognised. Experimental rodent models of TBI have demonstrated an increase in IL-6 gene expression in the brain within 1 h following injury [13], peak protein levels at 2 to 8 h after injury and levels in CSF peaking at 2 to 5 h [10]. Numerous clinical studies have demonstrated the upregulation of various inflammatory mediators within the blood of TBI patients, including IL-6 [14, 15]. Therefore, IL-6 potentially fulfils the essential criteria of a biomarker: it is present in body fluids, is detectable by existing assays and is associated with damage to a specific tissue [16].

Whilst there is ample evidence of detectable IL-6 release post-TBI, its relationship with clinical outcomes remains unclear. In this study, we systematically reviewed the literature to identify the value of IL-6 as a clinical biomarker in predicting outcomes in TBI patients.

## Methods

This study was performed in accordance with the Preferred Reporting Items for Systematic Reviews and Meta-Analyses (PRISMA) Statement [17]. The protocol for this systematic review is registered on the PROSPERO database (Reference: CRD42021271200).

### Literature search

**Fig. 1:**
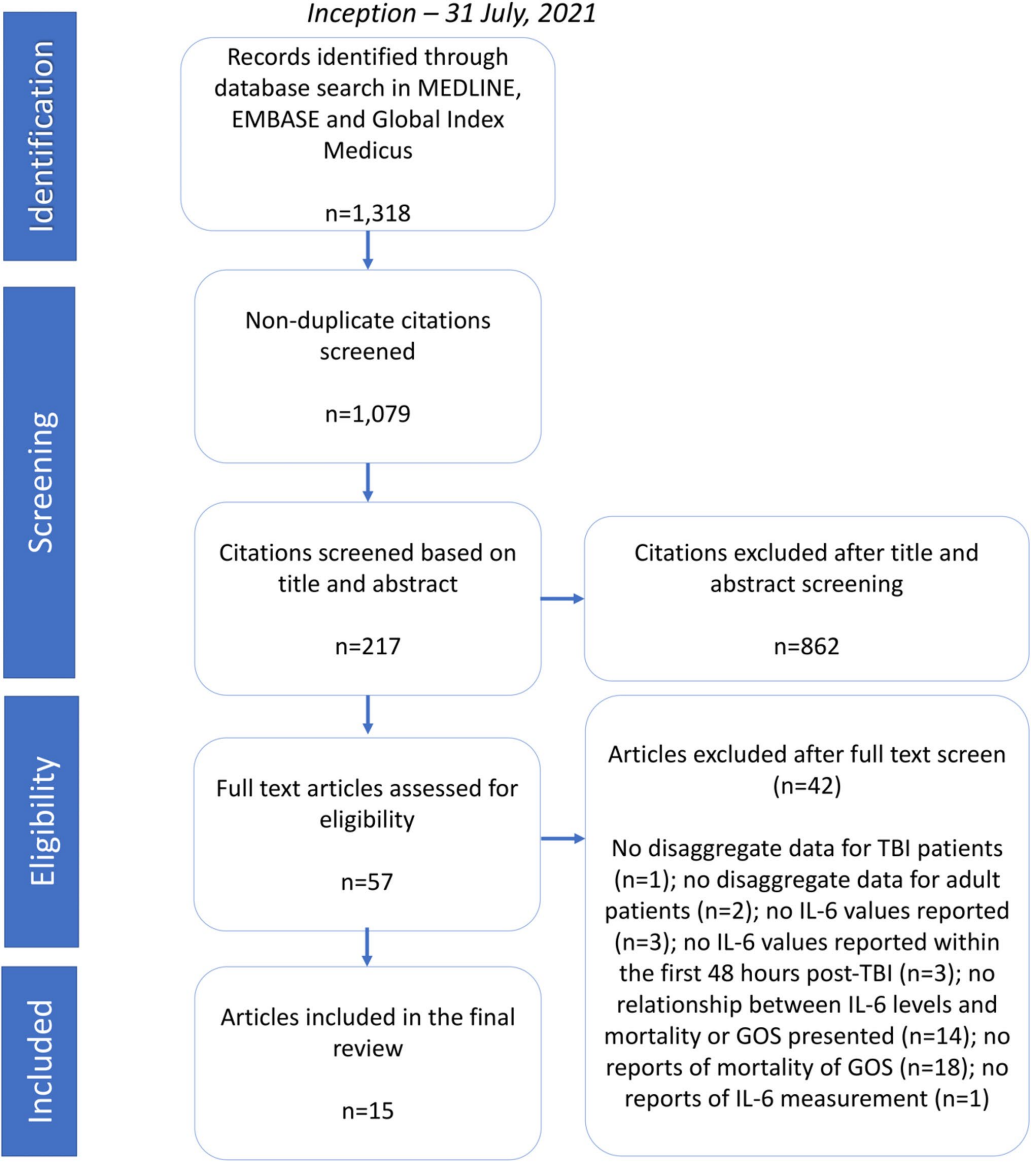
PRISMA flow chart of the source selection process. TBI, traumatic brain injury; GOS, Glasgow Outcome Scale; IL-6, interleukin-6

A multi-database (MEDLINE, Embase, Global Health) literature search was performed from the 1st of January 1946 to the 31st of July 2021 **(**Fig. 1**)**. The search strategy used variants and combinations of search terms related to IL-6, TBI, serum, plasma, cerebrospinal fluid and microdialysis (Supplementary Table 1). The bibliographies of included studies were screened for further relevant studies.

### Inclusion and exclusion criteria

Studies fulfilling the following criteria were included: (i) an exclusive diagnosis of brain injury of traumatic aetiology; (ii) patient age of 16 years or above; (iii) patients with IL-6 levels measured within 48 h post-TBI and reported details regarding body tissue/fluid (serum, CSF, or intraparenchymal), time of measurement and method of quantification; and (iv) reported clinical outcome. Exclusion criteria included: (i) significant life-threatening extracranial injuries; (ii) studies on animals only; (iii) abstracts, conference presentations, editorials and expert opinions; and (iv) articles not written in English.

### Study selection

The online literature management system Rayyan was used for study selection following the exclusion of duplicate texts [18]. Two authors (SZYO, MH) independently screened the titles, abstracts and full texts of the identified articles based on the pre-defined selection criteria (Fig. 1). Any disagreement between the two reviewers’ decisions prompted further discussion, with persisting conflicts resolved by MZ.

### Data extraction

The following variables of interest were extracted: number of patients, age, gender, GCS on admission, severity of TBI (‘mild’ was defined as GCS 14–15, ‘moderate’ 9–13 and ‘severe’ 3–8) [19], mechanism of injury, imaging findings, management (surgical or medical), body tissue/fluid used to measure IL-6 (serum/plasma, CSF or microdialysis), time point of IL-6 measurement, IL-6 levels and clinical outcomes (mortality and/or functional outcome with time point). The Glasgow Outcome Scale (GOS) was reported as ranging from 1 (death) to 5 (full recovery) or dichotomised into favourable/good (GOS 4–5) and unfavourable/poor (GOS 1–3) outcomes. Studies reporting modified Rankin scale (mRS) rather than GOS were dichotomised into favourable and unfavourable outcomes in a similar fashion and pooled with papers reporting GOS. The time point of outcome reporting was divided into short- (up to 1 month or hospital discharge) and long-term (over 1 month) follow-up periods.

### Risk of bias

All included studies were independently assessed for risk of bias using the Risk of Bias in Non-randomised Studies of Interventions (ROBINS-I) tool [20] by three authors (SM, NP, GP) independently, and any conflicts were resolved by RS.

### Statistical analysis

Eligible studies were evaluated for the possibility of a meta-analysis regarding the value of IL-6 in serum/CSF/brain parenchyma as a prognostic marker. However, heterogeneity in methodologies and statistical reporting rendered such meta-analyses inappropriate or impossible (further details below). Therefore, a synthesis without meta-analysis was reported [21].

## Results

### Study characteristics

Fifteen studies met the selection criteria, all representing cohort studies of prospective (*n* = 11; 73.3%), retrospective (*n* = 3; 20.0%) or unspecified (*n* = 1; 6.7%) design. The studies and their patient populations are detailed in Table 1. Five studies (33.3%) were published between 2000 and 2009, nine (60.0%) between 2010 and 2019 and one (6.7%) in 2021. The majority of studies had a moderate risk of bias (*n* = 8; 53.3%). Five studies (33.3%) were deemed to have serious risk, and two (13.3%) were deemed low risk. Details of the risk of bias assessment can be found in Supplementary Table 2.

**Table 1:** Summary of included studies analysing the prognostic value of interleukin-6 levels in patients with traumatic brain injury after a systematic review of the literature

Author, year	Location (period)	No. of hospitals/patients	Age (years), %age male	Mechanism of injury, admission GCS	Radiological findings	Management	IL-6 sampling location, timepoint	Outcome parameter(s), timepoint	ROBINS-I score
Pleines et al., 2001 [22]	Switzerland (NR)	1/13	35.9 (mean), NR	NR, GCS ≤8–100%	CC and ‘mass lesion’: 7 CC and diffuse injury: 6	EVD: 100% ICP monitoring: 100% Mass lesion evacuated: 5	Blood and CSF Daily for 14 days	GOS, ‘between 3 and 6 months’	Moderate
Singhal et al., 2002 [23]	Canada (NR)	1/36	34.4 (mean)/range 17–68 25 (69.4%) males	NR, GCS 3–8 (range) Median GCS-5	NR	EVD: 100% Some patients underwent craniotomy for decompression of mass lesion: not further specified ICU: 100%	Blood and CSF Peak concentrations during the initial post-TBI period	Stratified GOS, 3 months	Moderate
Suehiro et al., 2004 [24]	Japan (NR)	1/5	58.6 (mean)/73 (median)/range 17–75 4 (80%) males	NR GCS: 5–8 (range) Mean GCS: 6.6 Median GCS: 6	SDH and CC: 3 SDH, EDH and CC: 1 DAI: 1	Surgery: 4 Conservative 1	Blood Days 0 and 1	Stratified GOS Discharge	Serious
Winter et al., 2004 [25]	UK (2000–2001)	1/14	43.1 (mean)/42.5 (median)/range 21–77 7 (50%) males	NR GCS: 5–14 (range) Mean GCS: 9.8 Median GCS: 9	ASDH: 2 DAI: 2 ASDH and CC: 1 ASDH and EDH: 1 ASDH and ICH: 1 CC and ICH: 1 CC and EDH: 1 ASDH, CC and EDH: 1 ASDH, CC and ICH: 1 CC, CDS and EDH: 1 CC: 1 EDH: 1	Craniotomy: 7 ICP monitoring: 100% ICU: 100%	Blood and microdialysis Daily for a maximum of 6 days	Stratified GOS, 6 months	Moderate
Venetsanou et al., 2007 [26]	Greece (NR)	1/75	50 (mean)/range 18–75 50 (66.7%) males	NR GCS >8: 40 GCS ≤8: 35	NR	NR	Blood Within 24 h post-TBI	Mortality, 30 days	Serious
Stein et al., 2011 [27]	USA (NR)	1/24	31.6 ± 13.2 (mean ± SD) 23 (95.8%) males	RTC: 11 Falls: 8 Assault: 2 ‘Unspecified’: 3 GCS: 6.3 ± 4.0 (mean ± SD)	NR	ICP monitoring: 100% Conservative in ICU: 100%	Blood and CSF Twice daily for a maximum of 7 days	Dichotomised GOSE, 6 months Mortality In-hospital	Moderate
Aman et al., 2012 [28]	Indonesia (2010–2011)	1/40	30.78 ± 15.43 (mean ± SD) 31 (77.5%) males	NR GCS ≤8: 17 (42.5%) GCS >8: 33 (57.5%)	EDH: 17 (42.5%) CC: 6 (15%) EDH and CC: 14 (35%) SDH: 2 (5%) CC and CL: 1 (25%)	Haematoma evacuation craniotomy: 32 (80%) Decompressive craniectomy: 7 (17.5%) Depressed fracture correction and debridement: 1 (2.5%)	Blood, preoperative and 1-day postoperative	Dichotomised GOS, 1 and 3 months	Moderate
Ferreira et al., 2014 [29]	Brazil (NR)	1/24	33.8 ± 13.8 (mean ± SD) 24 (100%) males	MVA: 9 (37.5%) Autopedestrian: 7 (29.2%) Falls: 3 (12.5%) Assault: 5 (20.8%) GCS: 5.4 (3.2–7.0) (mean (25–75% quartiles))	NR	Craniotomy: 16 (66.7%) ICU: 100%	Blood, daily for 3 days	GOS (mean ± SD) Discharge from ICU Mortality NR	Moderate
Nwachuku et al., 2016 [7]	USA (NR)	1/32	31.0 ± 16.0 (mean ± SD), 22 (68.8%) males	MVA: 21 Falls: 5 MCA/ATV: 3 MV/Ped: 3 GCS: 6.0 ± 1.6 (mean ± SD)	Multicompartmental haemorrhage: 13 SAH: 6 SDH: 4 SAH/SDH: 2 CC: 2 EDH: 1 IPH: 1 DAI: 1 No bleed: 1 Cerebral oedema: 1	ICU: 32 (100%) EVD: 32 (100%) Decompressive operative management NR	CSF, daily for a maximum of 5 days	Stratified GOS, GOS (mean ± SD) and mortality, 6 months	Moderate
Deepika et al., 2018 [30]	Bangalore, India (NR)	1/89 (10 lost to follow-up)	34.9 ± 11.1(mean ± SD) 76 (85.4%) males	NR GCS: ≤8–100% Mean GCS: 6.3	Mass lesions: 71 Obliteration of the third ventricle: 67 SAH: 61 EDH: 27 Diffuse injuries: 18 Petechial haemorrhages: 12	Mass lesions evacuated: 70 No ICP monitoring	Blood, days 1, 3 and 10 post-injury	Dichotomised GOSE, 6 months	Moderate
Feng et al., 2018 [31]	Changxing, China (2013–2016)	1/102	35 (median)/range: 18–74 58 (56.9%) males	NR GCS ≤8: 100% Median GCS: 6	Midline shift >5 mm: 45 tSAH: 59	NR	Blood, admission	Mortality, 30 days	Serious
Lewis et al., 2019 [3]	USA (2013–2015)	1/76	47.8 ± 21 (mean ± SD) 60 (78.9%) males	NR GCS ≤8: 7 (9.2%) GCS >8: 69 (90.8%)	Intraventricular haemorrhage: 15 (19.7%)	NR	Blood, 24–48 h after TBI	Dichotomised modified Rankin Scale (converted to GOS) NR	Moderate
Shao et al., 2019 [32]	Hebei, China (2015–2016)	1/40	24 males; mean age: 47 16 females: mean age 52	RTC: 18 ‘Heavy impact’: 10 Fall from height: 7 ‘Motion impact’: 5 GCS: 8–12 (range)	NR	Operative treatment: 100%	Blood, pre-operatively, <24 h after admission	GOS 1 year	Serious
Zhang et al., 2019 [33]	China (2014–2018)	1/102	36 (25–53) (median (IQR)) 64 (62.7%) males	NR GCS <9: 102 (100%) GCS 5 (4–6) (median (IQR))	tSAH: 61 (59.8%) Mass lesions: 54 (52.9%) Abnormal cisterns: 46 (45.1%) Midline shift >5 mm: 42 (41.2%)	NR	Blood, at admission (<6 h after injury)	Dichotomised GOS and mortality, 6 months	Low
Kazakova et al., 2021 [34]	Bulgaria (2017–2018)	1/27	50 ± 17 (mean ± SD) 24 (88.9%) males	NR GCS <9: 27 (100%) GCS 6.0 (4.0: 6.5) (median (IQR))	Cerebral oedema: 27 (100%) SDH: 9 (29.03%) Varied combinations of multiple intracranial haemorrhages: 8 (25.8%) ICH: 5 (16.12%) CC: 4 (12.9%) tSAH: 3 (9.67%) EDH: 2 (6.45%)	Conservative in ICU: 27 (100%)	Blood and CSF, 24th hour after TBI	Mortality, 6 months	Low

*GCS*, Glasgow Coma Scale; *IL-6*, interleukin-6; *ROBINS-I*, risk of bias in non-randomised studies of interventions; *NR*, not reported; *CSF*, cerebrospinal fluid; *GOS*, Glasgow Outcome Scale; *GOSE*, Extended Glasgow Outcome Scale; *SD*, standard deviation; *ICP*, intracranial pressure; *tSAH*, traumatic subarachnoid haemorrhage; *SDH*, subdural haemorrhage; *aSDH*, acute subdural haemorrhage; *EDH*, epidural haemorrhage; *DAI*, diffuse axonal injury; *CC*, cerebral contusion; *CL*, cerebral laceration; *CDS*, compound depressed skull fracture; *ICH*, intracerebral haematoma; *IPH*, intraparenchymal haemorrhage; *RTC*, road traffic collision; *ICU*, intensive care unit; *EVD*, external ventricular drainage; *MVA*, motor vehicle accident; *MCA*, motorcycle accident; *ATV*, all-terrain vehicle; *MV/Ped*, pedestrian struck by motor vehicle; *IQR*, interquartile range

### Patient cohort

Across the fifteen studies, 699 patients were reported. In studies reporting sex of patients (*n* = 14, 686 patients), 71.7% were male. Among those reporting mean age (*n* = 13, 495 patients), the pooled mean was 40.8 years. Of studies reporting the age range of included patients (*n* = 8, 293 patients), the overall range was between 16 and 83 years. In studies reporting TBI severity (*n* = 13, 635 patients), 496 (78.1%) patients sustained a severe TBI and 139 (21.9%) sustained mild/ moderate TBI. Two studies reporting patients with mild and moderate TBI did not distinguish between the patients in the mild and moderate categories [35, 36]. Of studies reporting mean GCS on admission (*n* = 6, 188 patients), the overall mean GCS of 188 patients was 6.4. Among those reporting the range of GCS on admission (*n* = 7, 299 patients), the pooled range was between 3 and 14. The mechanism of injury was sparsely reported, being described in only four studies. Of the 120 patients reported in these studies, the most common mechanism of injury was road traffic collisions (RTC) (*n* = 72, 60.0%). RTC included motor vehicle (*n* = 30, 25.0%), auto-pedestrian (*n* = 10, 8.3%) and motorcycle accidents (*n* = 3, 2.5%). Other mechanisms of injury cited were falls (*n* = 23, 19.2%), ‘heavy impact’ (*n* = 10, 8.3%), assault (*n* = 7, 5.8%) and ‘motion impact’ (*n* = 5, 4.2%) [37]. The mechanism of injuries of three patients (3.8%) was unspecified. Of the 23 falls that occurred, 7 were stated to be ‘high falling’ [37]; the remaining were unspecified.

### Radiological findings

Ten studies (500 patients) described the neuro-radiology findings in patients sustaining TBI. Both computed tomography (CT) and magnetic resonance imaging (MRI) findings were reported. The most commonly cited finding was traumatic subarachnoid haemorrhage (SAH) (*n* = 190; 38.0%), followed by ‘mass lesion’ (*n* = 132, 26.4%), ‘midline shift >5mm’ (*n* = 87, 17.4%), epidural haemorrhage and obliteration of third ventricle (*n* = 67, 13.4% each), cerebral contusions (*n* = 51, 10.2%) and abnormal cisterns (*n* = 46, 9.2%). Other radiological findings were cerebral oedema (*n* = 28, 5.6%), subdural haemorrhage (SDH) and diffuse injury (*n* = 26, 5.2% each), intraventricular haemorrhage (*n* = 15, 3.0%), multicompartmental haemorrhage (*n* = 13, 2.6%) and petechial haemorrhages (*n* = 12, 2.4%). Less commonly cited findings were five (1.0%) patients with intracerebral haematoma, two (0.4%) with a mixed diagnosis of SAH/SDH and one (0.2%) patient each with a compound depressed skull fracture, cerebral laceration and intraparenchymal haemorrhage. One study reported eight patients (2.6%) with ‘varied combinations of multiple intracranial lesions’ [38]. Of note, several patients had more than one finding reported—details can be found in Table 1.

### Management

Eleven studies (344 patients) described the management of patients. Overall, 231 patients (67.2%) received operative management whilst the remainder were treated non-operatively. Craniotomy was the most common operative procedure performed (*n* = 55, 16.0%), followed by insertion of an external ventricular drain (EVD) ± mass lesion evacuation (*n* = 49, 14.2%), decompressive craniectomy (*n* = 7, 2.0%) and depressed fracture elevation and debridement (*n* = 1, 0.3%). Two studies, which reported a total of 75 patients (21.8%) who had a mass lesion evacuated, did not specify whether a craniotomy or craniectomy was performed [32, 39]. A further two studies, reporting a total of 44 patients (12.8%) who underwent surgical management, did not specify the procedure performed [33, 37].

Intracranial pressure (ICP) monitoring was performed in 119 patients (34.6%), whilst conservative management was delivered in an intensive care unit (ICU) in 45.6% (*n* = 157) of all patients reported. One patient (0.3%) was treated conservatively; however, no further details on management were given [33]. Of the 106 patients who underwent ICP monitoring, 36 were stated to possibly have had further intervention with ventricular drainage, intravenous mannitol and hyperventilation (in order of preference), if the intracranial pressure was greater than 25 mmHg for longer than 10 min [40].

### IL-6 analyses

Nine studies (594 patients) reported IL-6 concentrations in serum/plasma [28, 29, 33, 35–37, 39, 41, 42], three (132 patients) reported CSF levels [22, 32, 40] and two reported both [25, 38]. One study reported brain parenchymal IL-6 levels obtained via cerebral microdialysis [43].

The technique used for the detection of IL-6 in fluids was enzyme-linked immunosorbent assay (ELISA) in 13 studies [28, 29, 32, 33, 35–40, 42, 43], multiplex bead array systems in two [25, 41] and the Meso Scale Discovery® electrochemiluminescence system in one [22].

The time point of serum IL-6 level measurement was at admission or within 24 h of admission in nine studies, but Aman et al. reported the IL-6 level immediately post-decompressive surgery [28] and Deepika et al. analysed IL-6 levels collected on days 1, 3 and 10 of admission [39]. Time points for CSF sampling were more varied. CSF sampling was via EVD inserted for intracranial pressure management in all but one study, in which lumbar puncture was performed [38].

Levels of IL-6 in serum or CSF were reported in picogrammes per millilitre in 13 studies, with the remainder reported in nanogrammes per millilitre [32, 37]. However, the group median value of 239 ng/mL [32, 37] reported by Shao et al.—equivalent to 239,000 pg/mL—is extremely high when compared to the rest of the studies [37]. We have assumed this is a typographical error and should therefore read ‘239 pg/mL’.

The descriptive statistics of IL-6 values reported in included studies are shown in Tables 2 and 3 for serum and CSF, respectively. Among studies reporting serum levels for the whole cohort of TBI patients within 24 h of admission (*n* = 6), four studies (164 patients) reported the mean IL-6 value (range of means 88–382.9 pg/mL) [28, 33, 36, 41] and four (187 patients) reported the median IL-6 value (range 11.8–239 pg/mL) [28, 33, 37, 42]. As shown in Table 2, there was no obvious cause for this variation in values seen with respect to the IL-6 detection assay. Studies reporting CSF IL-6 levels varied significantly in their methodology for sample collection and data presentation (Table 3).

**Table 2:** Summary of studies investigating the use of serum interleukin-6 (IL-6) as a predictor of outcome

Study	Method of IL-6 analysis	IL-6 detection assay	Whole cohort's IL-6 values	Values reported in a comparison of outcome groups	Clinical outcome parameter(s), timepoint	Statistical analysis method	Finding(s)
Suehiro et al., 2004 [24]	Days 0 and 1 after admission levels presented	ELISA	Mean 382.9* (SD 378.7*) Median 163* (IQR 104.5–634*) Range 69–971	Individual patient (*n* = 7) values on day 0 and day 1	Discharge GOS	Not analysed statistically	No obvious pattern detectable from raw figures
Venetsanou et al., 2007 [26]	Single level sampled within 24 h after admission	ELISA	Mean 88 (SEM 16.3–SD 141.2*)	No values disaggregated by outcome group were reported aside from within a bar chart	30-day mortality	*t*-test	Significantly higher IL-6 values in non-survivors than survivors (*p* < 0.001)
Stein et al., 2011 [2]	Daily sampling for 7 days from admission—data presented for admission level and 7-day median levels	Multiplex Bead Array	7-day median 60.5 (IQR 29.9–149.9), range 4.3–13,706	Median admission and 7-day values	6-month GOS	*t*-test	Admission medians: 122.2 (good outcome) vs 104.9 (poor outcome), *p* = 0.9, 7-day median: 89.6 (good) vs. 104.3 (poor), *p* = 0.4
Aman et al., 2012 [28]	Admission and immediate post-operative IL-6 levels were presented. Only post-operative levels were analysed with respect to the outcome	ELISA	Admission mean 269.9 (SD 521.8), median 76.7 (range 0.08–2734.7) Post-operative mean 79.3 (SD 92.8), median 35.6 (range 5.1–353.2)	Numbers of patients with IL-6 values greater than or less than 100—no reason given for the use of this cut point	3-month GOS	Fisher’s exact test (for an association between categorical variables—post-operative IL-6 level vs. GOS)	Significantly more patients with ‘low’ IL-6 in favourable outcome group (93.5%) than in unfavourable outcome group (6.5%), *p* = 0.016
Ferreira et al., 2014 [29]	Admission to ICU and 24-h IL-6 levels	Cytometry bead-based assay	Admission mean 218.8 (SEM 56.5–SD 276.5*)	No values disaggregated by outcome group reported aside from within a bar chart	Short-term mortality	Mann-Whitney *U* test to compare the distribution of IL-6 levels at admission and 24 h timepoints, between survivors and non-survivors	Significantly higher IL-6 levels in non-survivors at admission and 24 h (no *p*-values)
Deepika et al., 2018 [30]	Sampled on days 1, 3 and 10 of admission	ELISA	NR	No values reported aside from within a line graph	6-month GOS	Multivariate logistic regression analysis for 6-month dichotomised GOS Parameters included heart rate variability metrics; cytokine panel including IL-6; IL-1β and IL-10; and the components of the CRASH2 predictive model (GCS, age etc.)	No significant predictive value of IL-6. Significant predictors identified: Low GCS and high IL-10 levels
Feng et al., 2018 [31]	Single sample at admission	ELISA	NR	Median (IQR) IL-6 values in survivors vs non-survivors	30-day mortality	Mann-Whitney *U* test	Significantly higher median IL-6 in fatalities (12.1 (8.8–15.5)) vs. survivors (8.8 (5.5–10.9)), *p* = 0.005
Lewis et al., 2019 [3]	Single sample at 24–48 h after injury	ELISA	NR	Median (IQR) IL-6 values in favourable vs. unfavourable outcome groups	Discharge mRS (dichotomised)	Multivariate logistic regression model for the predictive value of cytokine panel after controlling for age and GCS	Significantly higher median IL-6 in unfavourable group (190 (126–661)) vs. favourable group (133 (79–244)), adjusted *p* < 0.05 Significant also for IL-10 and RANTES levels
Shao et al., 2019 [32]	Single sample at 1 h prior to decompressive surgery and less than 24 h after admission	ELISA	Median 239 ‘ng/mL’ [sic]	Cohort split into two groups based on whether the IL-6 level was above or below the median value	1-year GOS	Cross-tabulations presented in paper—high/low IL-6 vs. GOS Fisher’s exact test performed on this data by the present authors (RS)	No significant association between the IL-6 category and dichotomised GOS—48.4% of favourable group had ‘high’ IL-6 vs. 55.6% of unfavourable group, *p* = 0.7
Zhang et al., 2019 [33]	Single sample at admission	ELISA	Median 11.8 (IQR 8.8–14.4)	No values disaggregated by outcome group	6-month mortality and dichotomised GOS	Receiver operator analysis; *z*-test comparison between IL-33 and other cytokines including IL-6	AUC (95% CI) for IL-6 in predicting: (i) 6-month mortality: 0.650 (0.549–0.741); (ii) unfavourable outcome: 0.587 (0.485–0.683) IL-6 performed significantly less well than IL-33 in these analyses, (i) *p* = 0.033 and (ii) *p* < 0.001, respectively
Kazakova et al., 2021 [34]	Samples taken on the 24th and 96th hours after trauma	ELISA	NR	Median (IQR) IL-6 in survivors vs. non-survivors	6-month mortality	Mann-Whitney *U* test	No significant difference in median IL-6 level at 24h in survivors (123 (IQR 71–187)) vs non-survivors (96 (35–159)), *p* = 0.846 96 h levels not presented or analysed because not significantly different from 24 h levels

*IL-10/-33*, interleukin-10/-33; *ELISA*, enzyme-linked immunosorbent assay; *SD*, standard deviation; *IQR*, inter-quartile range; *SEM*, standard error mean; *GOS*, Glasgow Outcome Scale; *NR*, not reported; *GCS*, Glasgow Coma Scale; *mRS*, modified Rankin Scale—a similar metric to GOS which can be dichotomised into good/favourable and poor/unfavorable outcomes; *ICU*, intensive care unit; *RANTES*, Regulated upon Activation, Normal T-Cell Expressed, presumably Secreted—aka CCL5, C–C chemokine ligand 5; *AUC*, area under (receiver operator) curve; *CI*, confidence interval. IL-6 levels are reported in picogrammes per millilitre unless otherwise stated. Values marked with an asterisk (*) are calculated by the present authors from figures presented in the original articles

**Table 3:** Summary of studies investigating the use of cerebrospinal fluid (CSF) interleukin-6 (IL-6) level as a predictor of outcome

Study	Method of IL-6 analysis	IL-6 detection assay	Whole cohort’s IL-6 values	Values reported in a comparison of outcome groups	Clinical outcome parameter(s), timepoint	Statistical analysis method	Findings
Pleines et al., 2001 [22]	Daily CSF collection for 14 days post-injury—averaged across 14 days	ELISA	Overall mean 188.9* Range of individual patient 14-day means 100–8200	Individual patient mean ± SEM	3–6-month GOS	Linear regression model for prediction of GOS	No significant predictive value of IL-6 levels
Singhal et al., 2002 [23]	Peak measured concentration	ELISA	Median 650 Range 130–7214	Median, range	3-month GOS	Non-parametric ANOVA (Dunn’s test) for IL-6 levels between GOS groups; Multiple regression model for GOS as a categorical outcome	Peak IL-6 levels increased with improving GOS (omnibus *p* = 0.024) Peak IL-6 level was an independent predictor of GOS (*p* = 0.026); along with age (*p* = 0.0072); pupillary abnormality (*p* = 0.021); presence of a ‘mass lesion’ (*p* = 0.023)
Stein et al., 2011 [27]	Twice daily collection for 7 days/until removed if sooner	ELISA	7-day median 352.6 (IQR 137.1–1354.7) Range 3.7–11,459.7	No disaggregated values reported	6-month GOS	Wilcoxon rank-sum test to compare the distribution of (i) admission IL-6 levels and (ii) averaged daily IL-6 levels with dichotomised GOS	No significant difference in either IL-6 metric between outcome groups
Nwachuku et al., 2016 [7]	Daily CSF collection for 5 days post-injury	Meso Scale Discovery	Day 5 mean 175.9 (95% CI 93.6–331.3)	Mean ± SD and median across days 1–5, by dichotomised GOS groups	6-month GOS	Kruskal-Wallis test	Significantly higher median IL-6 in unfavourable outcome group (1899) vs. favourable outcome group (639), *p* = 0.03
Kazakova et al., 2021 [34]	Samples taken on the 24th and 96th hours after trauma	ELISA	NR	Median (IQR) IL-6 in survivors vs. non-survivors	6-month GOS	Mann-Whitney *U* test	No significant difference in median IL-6 level at 24 h in survivors (186 (IQR 180–195)) vs. non-survivors (189 (178–247)), *p* = 0.662 96 h levels not presented or analysed because not significantly different from 24 h levels

*SEM*, standard error mean; *GOS*, Glasgow Outcome Scale; *SD*, standard deviation; *IQR*, interquartile range. IL-6 levels are reported in picogrammes per millilitre unless otherwise stated. Values marked with an asterisk (*) are derived by the present authors from figures presented in the original articles

Three studies reported paired CSF and serum samples [25, 32, 38]. All of these demonstrated higher concentrations of IL-6 in CSF than in serum in the TBI population; however, the graphical representations in Pleines et al.’s paper indicate that the temporal trends in concentration are mirrored between CSF and serum [32].

The study by Winter et al. utilised cerebral microdialysis for the measurement of parenchymal IL-6 concentrations in patients with severe TBI who were intubated and ventilated in the ICU [43]. They inserted the probe into the left frontal region in all patients unless this was the site of primary traumatic pathology, in which case the right frontal region was used. They placed the microdialysis catheter purposefully away from the region of primary pathology to monitor cytokine dynamics related to ‘diffuse-type damage’ rather than areas with parenchymal contusion/haematoma visible on neuroimaging. The dialysate was sampled three to four times daily for a maximum of 6 days (shorter if the patient was extubated prior to this) [43].

### Clinical outcome reporting

Among the fifteen studies, five reported short-term (up to 1 month post-injury) outcomes [29, 33, 35, 36, 41] and the remainder reported longer-term follow-up (maximum 1 year).

GOS was used as an outcome measure in twelve studies, whilst mortality alone was reported in five [29, 36, 38, 41, 43]. In total, 368 patients were included in all studies that reported mortality (*n* = 10), of whom 125 died (34.0%) [22, 29, 32, 33, 36–38, 40, 41, 43]; 366 patients were included in studies reporting long-term GOS (*n* = 8), of which 205 patients had a good outcome (56.0%) [22, 25, 28, 32, 37, 39, 40, 42].

### IL-6 levels in the blood as a predictor of clinical outcomes

Eleven included studies examined the relationship between serum IL-6 concentration and clinical outcome in TBI patients [25, 28, 29, 33, 35–39, 41, 42] (Tables 1 and 2**)**. Among these, three studies (121 patients) reported GOS at hospital discharge or 30 days post-injury [28, 33, 35]; four studies (206 patients) reported mortality within 30 days of injury [29, 33, 36, 41]; five studies (285 patients) reported long-term GOS [25, 28, 37, 39, 42]; and two studies (129 patients) reported long-term mortality [38, 42].

In studies reporting short-term outcomes [28, 29, 33, 35, 36, 41], one study presented five patients with no mortality and no discernible predictive value of admission serum IL-6 levels with respect to outcome [33]. Four other papers all showed significantly higher serum IL-6 levels shortly after admission to the hospital in those patients with poor outcomes [35] or who died [29, 36, 41] compared to those with favourable outcomes or who survived, respectively. However, in the short-term outcomes data presented by Aman et al. [28], there was no significant association between post-operative IL-6 and 1-month GOS.

One study [35] demonstrated that the median serum IL-6 level at 24–48 h after admission was significantly higher in those with poor outcomes at discharge (*n* = 76; IL-6 190 vs. 133 pg/mL). The reported median (IQR) length of stay in hospital for the 76 patients was 15.15 days (7.9–24). This significant finding remained after controlling for age and initial GCS score in a multivariate logistic regression model, using IL-6 concentration as a continuous variable (adjusted *p* < 0.05, no odds ratios reported). Similarly, other studies demonstrated higher serum IL-6 levels at admission in patients who died compared to survivors at 30 days [29, 36] and at the time of ICU discharge [41].

Six studies reported the relationship between serum IL-6 levels and long-term outcomes [25, 28, 37–39, 42]. Aman et al. demonstrated that an immediate post-operative serum IL-6 level greater than 100 pg/mL was associated with poor outcomes at 3 months (*p* = 0.016) [28]. However, other studies showed no significant association between serum IL-6 measurements taken within 24h of admission and extended GOS [25] or mortality [38], respectively at 6 months. One study analysed patients based on whether their serum IL-6 level within 24 h after admission was above or below the group (*n* = 40) median. There was no significant association between this metric and dichotomised GOS at 1 year [37]. Deepika et al. performed a multivariate logistic regression analysis for predictors of dichotomised GOS at 6 months, including a cytokine panel, heart rate variability parameters and the validated predictors of outcome from the CRASH2 prognostic model [5] as putative predictors. They found no significant effect of IL-6 in this model [39]. A further study presented a receiver operator analysis for the prognostic value of IL-6 with respect to mortality and dichotomised GOS at 6 months, reporting the area under the receiver operator curves (AUC) only. The AUC for IL-6 concentration at hospital admission was 0.650 (95% CI 0.549–0.741) for mortality and 0.587 (95% CI 0.485–0.683) for GOS [42].

### IL-6 levels in CSF as a predictor of clinical outcomes

Five included studies reported the association between IL-6 levels in patients’ CSF and their clinical outcome [22, 25, 32, 38, 40] (Tables 1 and 3). All of these studies reported long-term outcomes—either GOS [22, 25], mortality [38] or both [32, 40].

Three studies utilised daily CSF collection, resulting in a composite measure of IL-6 concentration as an average of all time points [22, 25, 32]. One study analysed CSF sampled 6 hourly for the first 5 days post-injury in their cohort of 32 patients. The median IL-6 value was significantly higher in the poor outcome group (*n* = 18) than the good outcome group (*n* = 14) (1899 vs. 639 pg/mL; *p* = 0.03) at 6 months. No multivariate analysis was performed in this study [22]. Another study involved CSF collection from 14 patients twice daily for a maximum of 7 days [25]. There was no significant finding when comparing the median daily CSF IL-6 levels to dichotomised GOS at 6 months [25]. The final study collected CSF daily for 14 days from 13 patients, and individual patients’ mean IL-6 values showed no significant correlation with their GOS at 3–6 months [32].

Two studies involved periodical sampling of CSF at longer time intervals [38, 40]. One study involved periodical sampling of CSF for a mean duration of 73.6 h, with the peak IL-6 level occurring at a mean of 36.1 h post-injury [40]. One of these demonstrated that patients with better clinical outcomes had significantly higher peak CSF IL-6 levels in comparison between GOS categories (omnibus *p* = 0.026; GOS 1 median IL-6 = 412 pg/mL vs. GOS 5 = 1650 pg/mL. This finding remained significant in multiple regression analysis, with other significant predictive factors being increasing age, presence of pupillary abnormality and the presence of a mass lesion [40]. In the study by Kazakova et al., CSF was sampled at 24 and 96 h post-injury in 27 patients. There was no significant difference in CSF cytokine concentrations (including IL-6) between the 24- and 96-h time points, and they found no difference in the median IL-6 concentration at 24 h between survivors and non-survivors (*p* = 0.662) [38].

### Parenchymal IL-6 levels in relation to clinical outcomes

One study demonstrated that patients who survived to 6 months post-injury had a significantly higher median peak IL-6 level in their brain parenchyma than those who died. Exact values were not reported, but graphical evidence [43] shows that the median IL-6 concentration in survivors was approximately 550 pg/mL, compared to around 100 pg/mL in non-survivors (*n* = 14, *p* = 0.04). No multivariate analyses were performed in this study with respect to IL-6 levels and outcome; however, they demonstrated no significant association between parenchymal IL-6 levels and initial GCS [43].

None of the included studies reported using the IL-6 levels measured to inform decisions about the clinical management of patients.

## Discussion

The life-threatening or life-altering nature of TBI and the societal cost of its aftermath make it a disease that requires new therapeutic options. Despite the predictive value of the GCS being described some decades ago, at present, there are no biomarkers known to predict patient outcomes in routine clinical use. Several markers have been suggested to be predictive of long-term outcomes in TBI patients, such as inflammatory mediators including IL-1β, IL-10, IL-33, TNF-α and IL-6 [32, 41, 42, 44–46]. Preliminary studies have also suggested a prognostic role for neuron- or glial cell-specific proteins, such as S100B, neurofilament light, neuro-specific enolase, myelin basic protein (MBP), glial fibrillary acidic protein (GFAP), phosphorylated axonal neurofilament subunit H (pNF-H), tau protein and ubiquitin carboxyl-terminal hydrolase L1 (UCH-L1). The presence of these molecules in the peripheral circulation implies traumatic blood-brain barrier disruption, perhaps providing an indication of an aspect of TBI severity [46, 47]; however, their robustness as tools for prognostication in TBI patients is yet to be defined.

Effective treatments are also lacking—whilst surgical decompression and neuro-protective measures to maintain cerebral perfusion pressure comprise the mainstay of acute management, evidence from pre-clinical studies suggests that the neuroinflammatory response is of critical importance in the brain’s recovery from TBI, thus representing a platform for the exploration of prognostic biomarkers as well as therapeutic targets [26]. Our systematic review focusses on the well-described pro-inflammatory cytokine IL-6, known to be important in inflammatory, infectious and neoplastic diseases of multiple organ systems [48–50] and previously shown to be produced in the brain in response to TBI [51]. We have systematically reviewed the literature published to date in order to assess the potential role of IL-6 as a prognostic biomarker for patient outcomes after TBI.

### Patient population

The patient cohort represented by these studies is largely representative of the severe TBI population in general. The average age of included patients was 40.8 years, with a significant male preponderance. This is consistent with the demographics of patients with TBI: commonly young male individuals [52, 53]. Most of the patients had a severe TBI, and the most common mechanism of injury was RTC. This differs from published literature on the epidemiology of TBIs, in which falls are the most common cause of injury [53]. However, mechanisms of injury were only reported by a minority of papers (see Table 1), and the preponderance of patients with severe TBI likely excludes those with mild/moderate TBI resulting from low-energy falls. Traumatic SAH was the most common finding reported on CT neuroimaging. Approximately one-third of the patients were managed with ICP monitoring. Of those who received surgical intervention, a quarter was reported to have undergone a craniotomy, although specific details of operative management were unspecified in several papers (Table 1).

### Key findings

Across the eleven studies that reported the relationship between serum IL-6 levels and their clinical outcome, five showed that a higher IL-6 concentration following TBI was associated with poorer outcomes [28, 29, 35, 36, 41], five showed no significant association [25, 33, 37–39] and one demonstrated the predictive ability of serum IL-6 levels with respect to long-term outcomes but did not report any specific values of IL-6 [42]. A very wide range of serum IL-6 values was reported, with group averages ranging from 11.8 pg/mL [42] to 382.9 pg/mL [33] and extreme values ranging from 0.08 pg/mL [28] to 13,706 pg/mL [25]. These discrepancies are not explained by differing IL-6 detection assays (Table 2) but may be due to the patient population, clinical management and/or sample collection practices. For example, when comparing the studies with the two most extreme values, the study with the largest value comprised only patients with conservatively managed severe TBI [25], whereas in the study with the smallest IL-6 value, more than half of the patients had mild/moderate TBI but 97.5% underwent decompressive surgery [28].

The six studies that analysed CSF IL-6 levels also had conflicting findings. Three studies found no association between CSF IL-6 concentrations and outcomes [25, 32, 38]. However, Nwachuku et al. demonstrated higher IL-6 levels in patients with poor outcomes [22], whereas Singhal et al. showed the opposite, which remained significant in multivariate analysis [40]. In comparing these two conflicting studies, the patient population is similar—all patients had a GCS of 8 or less on admission and were conservatively managed in ICU. However, the reporting of IL-6 differed, with one study measuring a composite of IL-6 measurements during the first 5 days post-injury [22], whilst the other measured peak serum IL-6 levels [40]. Therefore, the association between CSF IL-6 levels and clinical outcomes in TBI requires further exploration. All of these studies collected CSF from patients with EVDs in situ other than one in which lumbar puncture was performed if no EVD was used [38]. They did not report the CSF IL-6 levels in the lumbar puncture patients separately from the EVD patients, and the median IL-6 values presented in this paper were within the range of CSF IL-6 values across the other four relevant papers (Table 3) [22, 25, 32, 40]. Only one of the studies utilising reported the rate of CSF infection in their cohort—6.25% in Nwachuku et al.’s study—however, they did not report whether the CSF IL-6 levels were significantly different in those patients with ventriculitis than those without [22].

One study analysed brain parenchymal levels of IL-6 in severe TBI patients using cerebral microdialysis [43]. They purposefully targeted a brain region that was anatomically distant from any focal injury. Thus, the cytokine profile represents the general microenvironment of the traumatised brain and is more likely to be reflective of brain pathology given the relative integrity of the blood-brain barrier. The results of this study were not in keeping with the otherwise prevailing notion that an exaggerated inflammatory reaction (evidenced by greater IL-6 production) is associated with a poorer outcome—whether this relates to differential IL-6 levels in brain parenchyma as compared to CSF/serum, or other factors such as patient selection, it is not possible to elucidate [43].

Eleven included studies described the clinical management of their patient cohort, and two specifically reported IL-6 levels obtained prior to any surgical intervention [28, 37]. However, no studies used the measured IL-6 concentrations in making decisions on patient management, and indeed, very little detail was given regarding the factors influencing management decisions generally. Only one study attempted to assess the impact of the surgical intervention itself on IL-6 levels—Aman et al. measured serum levels at admission and 1 day post-operatively in their cohort of 40 surgically managed patients. They demonstrated a significant reduction in serum IL-6 levels after surgical intervention (mean reduction of 190.6 pg/mL, *p* = 0.001); however, the timing of surgery was not specified to allow comparison with other studies based on temporal trends alone [28].

The temporal dynamics of IL-6 levels were inconsistently reported among studies sampling at multiple time points. Pleines et al. showed the IL-6 concentrations in CSF and serum for 14 days after injury, showing an initial drop from admission to day 5 before a second peak in IL-6 concentration on day 6 followed by a steady reduction thereafter [32]. However, no studies examined for any association between IL-6 dynamics and outcome—e.g., whether the rate of change in IL-6 concentration from one time point to another is predictive of poor outcome. However, two studies demonstrated data that imply a potential effect of this. Ferreira et al. showed graphically that patients who died had steadily increased serum IL-6 levels at 0, 24 and 72 h, whereas a biphasic response was seen in survivors with a reduction from 0 to 24 h then an increase at 72 h to levels greater than baseline. The absolute values at 0 and 24 h were higher in non-survivors than survivors [41]. Stein et al.’s study utilising twice-daily serum collection over 14 days also pointed to the possible importance of IL-6 dynamics. In their cohort of patients with favourable outcomes, the median admission IL-6 concentration was 122.2, with the median for the whole 14-day period being 89.6, suggesting an average reduction compared to admission levels. However, in the unfavourable outcome group, the admission median was 104.9 pg/mL, whilst the 14-day median was 104.3 pg/mL, implying higher levels for a longer period in the unfavourable group than in the favourable group. The absolute values were not significantly different between outcome groups, but the dynamic effect was not analysed statistically [25].

Although several included studies analysed more cytokines than IL-6 alone, none analysed the relative concentrations between them—for example, whether the ratio of ‘pro-inflammatory’ IL-6 to ‘anti-inflammatory’ IL-10 is of prognostic significance.

### Risk of bias assessment

As shown in Table 1 (further details in Table S1), six of the fifteen included studies suffered from a serious risk of bias with respect to the relationship between IL-6 levels and clinical outcomes. This arose from a lack of assessment or adjustment for potential confounders that were likely to predict clinical outcomes, such as initial GCS. For example, two studies with significant findings demonstrated that IL-6 levels independently predicted clinical outcome [35, 40], and another showed that serum IL-6 levels were associated with survival but not with initial GCS [36], but four other studies reported significant findings without any adjustment for relevant confounders [22, 28, 29, 41]. Indeed, one study demonstrated that serum IL-6 levels at admission and immediately post-operatively were significantly associated with both GCS at admission and 1 week post-injury [28], whilst Ferreira et al. found a significant association between admission serum IL-6 level and the APACHE-II score used universally in intensive care medicine mortality prediction [41]. Furthermore, in the study by Deepika et al., a graphical representation of the univariate association between serum IL-6 values and 6-month GOS suggested significantly higher levels in the unfavourable outcome group at days 3 and 10. However, this effect was not significant after adjustment for several other cytokines, heart rate variability parameters and the previously established CRASH2 predictors, demonstrating the potential pitfalls of univariate comparisons in this setting [39].

### Implications

Although the literature published to date is limited in breadth and contains some inconsistencies in findings, it is clear that TBI results in IL-6 production in the brain, and that exaggerated IL-6 production following TBI may be predictive of poor clinical outcomes. IL-6 is an important cytokine in the inflammatory response throughout the body [54], as well as having functions in bone remodelling and muscle regeneration [31, 55]. Outside of the central nervous system, IL-6 is expressed as part of the innate immune response by immune cells—primarily macrophages. However, in the brain, IL-6 production is not limited to microglia/macrophages but has also been shown to occur in astrocytes and even neurons [11, 12, 56].

The cellular effects of IL-6 signalling are mediated through its specific receptor, IL-6 receptor α (IL-6Rα), albeit with differing downstream intracellular signalling depending on whether the membrane-bound or soluble form is activated [24]. It has conventionally been thought of as a purely pro-inflammatory cytokine, but there is evidence that it can also have anti-inflammatory effects, depending on the receptors/cells it acts on as well as the relative concentrations of other cytokines in the microenvironment [24, 30, 57].

The importance of the neuroinflammatory response following TBI has been increasingly recognised over recent years, with evidence that a degree of neuroinflammation is required for clearance of debris, as well as enhancing post-traumatic cortical neurogenesis, but that other aspects of neuroinflammation prohibit new neurons from maturing and performing brain repair [7, 26]. A key mediator in the generation and maintenance of the neuroinflammatory response is High Mobility Group Box 1 (HMGB1), a non-histone DNA-binding protein that serves as a DAMP, indicating cell necrosis in the immediate post-injury period. However, HMGB1 is thought to be actively secreted by immune cells thereafter, resulting in the perpetuation of the inflammatory process and a limitation of neural regeneration and functional recovery [7]. Binding of HMGB1 to its receptors (receptor for advanced glycation end products (RAGE); and toll-like receptor (TLR) 2/4) results in complex intracellular signalling cascades converging on the transcription factor nuclear factor kappa B (NFκB), which in turn upregulates transcription of various pro-inflammatory cytokines including TNF-α, IL-1β and IL-6 [58].

The exact effector molecules in this series of events that might be successfully targeted in order to improve outcomes after TBI are as yet unknown. Ample pre-clinical evidence exists to suggest that manipulation of the neuroinflammatory response can provide functional benefits [7], but to date, no in-human clinical trials have been able to demonstrate such effects [23, 26, 34, 59].

Whilst the exact role of IL-6 in the context of post-TBI neurogenesis, secondary brain injury and functional recovery is yet to be defined, it is clear is that it is highly upregulated in TBI and is detectable at much higher concentrations in both the blood and CSF than in the physiological condition [29, 33, 36, 37, 41, 45]. CSF levels were consistently higher than serum levels; however, an analysis of whether the difference between CSF and serum levels is predictive of outcome in itself (perhaps reflecting the degree of blood-brain barrier disruption) is absent to date. Further study is required to elucidate the temporal trends of IL-6 release after TBI and their implication for prognosis—for example, it might be the case that the initial release of IL-6 is proportional to the severity of the injury, whereas later release after a few days is a marker/promoter of neural regeneration and repair which would therefore favour better outcomes.

This systematic review demonstrates the limited evidence to support the notion that the degree of IL-6 production in the injured brain predicts patients’ capacity for recovery, which hypothetically relates to the degree of neuroinflammation and the resulting ability for neural regeneration.

The ideal biomarker for prognostication in TBI patients would be readily accessible, predictive within 24 h after hospital admission and both sensitive and specific for the outcome. IL-6 concentration in serum is therefore attractive, given that all TBI patients will have blood samples taken shortly after hospital admission, and six studies included in this systematic review demonstrate the promise of serum levels within a short timeframe [28, 29, 35, 36, 41, 42]. However, this review purposely focussed on a population of patients without significant extra-cranial injuries and the relevance of serum IL-6 concentrations in the context of multiple trauma, including TBI, requires exploration given the ubiquitous nature of IL-6 in the inflammatory response. To date, one study has explored the impact of the surgical intervention itself on circulating IL-6, indicating no significant additional release caused by surgical trauma [28]. CSF IL-6 levels are likely to be more specific to TBI outcomes in the context of multiple trauma; however, CSF is not as readily available for analysis as blood is, and hence any clinical utility would probably be limited to the severe TBI population undergoing EVD insertion.

Whilst biomarkers for the prediction of clinical outcomes will no doubt be useful to clinicians managing TBI patients, there is an urgent need for novel therapies in this population. Monoclonal antibodies targeting IL-6 such as tocilizumab are licensed for use in autoimmune diseases [50] and have been shown to be useful in COVID-19 with defined and acceptable side-effect profiles [49]. Therefore, modulation of IL-6 signalling is possible and could be explored as a therapeutic option in the context of TBI.

### Limitations

This systematic review is limited by the relatively small body of literature published to date on the topic, representing 699 patients in total, with several papers suffering from a serious risk of bias—therefore, the literature would benefit from large, prospective studies in this field. Any such studies must have clear case selection and employ robust statistical processes for controlling for important confounders, including initial GCS. Within the fifteen included papers, heterogeneity in methods and statistical reporting between studies excluded the possibility of meta-analysis (Tables 2 and 3). In particular, the practices of IL-6 sampling and data presentation varied greatly between studies, perhaps reflecting the developing nature of the field. Several studies utilised either the peak measured IL-6 concentration in blood/CSF or else the average IL-6 concentration over several days following the injury. However, the dynamics of IL-6 production and release are incompletely defined in human TBI.

Important common complications associated with traumatic brain injury and critical care such as EVD-associated ventriculitis and sepsis were also under-reported. For example, only two cases of ventriculitis were reported in our review of 699 patients, compared to typical rates of 5–15% reported in the wider literature [60–62]. Such complications have previously been shown to significantly impact IL-6 concentrations in bodily fluids and therefore represent important confounders [60, 63].

From the point of view of prognostication, the ideal circulating biomarker would be predictive of the outcome either at the point of hospital admission or at least within 24 h. Indeed, pre-clinical studies have demonstrated upregulation of IL-6 production within a few hours after injury [10, 13]. Therefore, the utility of IL-6 levels in the blood, CSF and/or parenchymal microdialysate in predicting later outcomes should be explored further. Lastly, we recognise that the included patients in the study predominantly had severe TBI (78.1%), thus the generalisability of our findings to the wider TBI population may be limited. The role of IL-6 in mild and moderate TBI remains unclear, and thus, warrants more prospective studies within this patient population. The mild/moderate TBI population has a better prognosis at baseline than that of the severe TBI population represented in this review. Therefore, future studies may benefit from employing a sliding dichotomy analysis with respect to GOS, to adjust for prognosis at baseline [64]. Studies in mild and/or recurrent TBI should also consider more subtle functional outcomes such as return to work, post-traumatic stress disorder (PTSD) and neurocognitive sequelae such as fatigue, and their relationship to IL-6 levels. One such example is the recent study by Rodney et al., indicating higher IL-6 concentrations in TBI patients suffering from long-term PTSD symptoms [27].

## Conclusion

This systematic review of the literature published to date regarding the prognostic value of IL-6 level concentration as a biomarker in TBI identified several papers with data suggestive of a useful role for the cytokine in this context. These studies suggest that exaggerated IL-6 secretion predicts poor outcomes. However, there is also limited evidence to the contrary, and heterogeneity between studies prohibited statistical meta-analysis. Large, prospective studies are required to confirm or refute these findings, and exploration of the importance of both IL-6 concentration dynamics and the relative concentrations of IL-6 with other cytokines would be prudent to study. Furthermore, the effects of pharmacological IL-6 modulation in this context should be explored in both pre-clinical and clinical studies.

## Supplementary Information

ESM 1 (DOCX 18 kb)

## Data Availability

Data are available on request to the corresponding author.

## Code availability

Not applicable to the present manuscript.
