# Supplementary material for: Interleukin-6 as a prognostic biomarker of clinical outcomes after traumatic brain injury: a systematic review
Source: Neurosurg Rev. 2022 Jul 6;45(5):3035–54. doi: 10.1007/s10143-022-01827-y (PMC9256073; doi:10.1007/s10143-022-01827-y)
Supplement: Supplementary file 1 — (DOCX 18 kb) [file 10143_2022_1827_MOESM1_ESM.docx]

**Table S1**. Details of literature search terms.

| 1 | IL6 OR IL-6 OR interleukin-6 |
| --- | --- |
| 2 | serum OR plasma OR microdialysis |
| 3 | trauma* |
| 4 | brain OR cerebral |
| 5 | injury OR injuries |
| 6 | TBI OR traumatic brain injur* |
| 7 | 3 AND 4 AND 5 |
| 8 | 6 OR 7 |
| 9 | 1 AND 2 AND 8 |

**Table S2**. Details of ROBINS-I scoring for included studies

| **Study, year** | **Confounding** | **Selection** | **Classification** | **Deviations from intention** | **Missing data** | **Outcome measurement** | **Results reporting** | **Overall** |
| --- | --- | --- | --- | --- | --- | --- | --- | --- |
| Pleines et al, 2001 | Moderate | Moderate | Low | Low | Low | Moderate | Low | Moderate |
| Singhal et al, 2002 | Moderate | Low | Low | Moderate | Low | Moderate | Low | Moderate |
| Suehiro et al, 2004 | Serious | Moderate | Low | Low | Low | Moderate | Low | Serious |
| Winter et al, 2004 | Moderate | Moderate | Low | Low | Low | Moderate | Low | Moderate |
| Venetsanou et al, 2007 | Serious | Low | Low | Moderate | Low | Low | Low | Serious |
| Stein et al, 2011 | Serious | Low | Low | Moderate | Low | Low | Low | Moderate |
| Aman et al, 2012 | Low | Low | Low | Low | Low | Moderate | Low | Moderate |
| Ferreira et al, 2014 | Serious | Moderate | Moderate | Low | Low | Low | Low | Serious |
| Nwachuku et al, 2016 | Serious | Low | Moderate | Low | Low | Low | Low | Moderate |
| Deepika et al, 2018 | Low | Moderate | Moderate | Low | Low | Moderate | Low | Moderate |
| Feng et al, 2018 | Serious | Low | Moderate | Low | Moderate | Low | Low | Serious |
| Lewis et al, 2019 | Low | Moderate | Moderate | Low | Low | Low | Moderate | Moderate |
| Shao et al, 2019 | Serious | Low | Low | Low | Low | Moderate | Low | Serious |
| Zhang et al, 2019 | Low | Low | Low | Low | Low | Low | Low | Low |
| Kazakova et al, 2021 | Serious | Low | Low | Low | Low | Low | Low | Serious |
